# Controversies in the Management and Followup of Differentiated Thyroid Cancer: Beyond the Guidelines

**DOI:** 10.1155/2012/512401

**Published:** 2012-12-30

**Authors:** Hala Ahmadieh, Sami T. Azar

**Affiliations:** Division of Endocrinology, Department of Internal Medicine, American University of Beirut Medical Center, 3 Dag Hammarskjold Plaza, New York, NY 10017, USA

## Abstract

Thyroid cancer is among the most common endocrine malignancies. Genetic and environmental factors play an important role in the pathogenesis of differentiated thyroid cancer. Both have good prognosis but with frequent recurrences. Cancer staging is an essential prognostic part of cancer management. There are multiple controversies in the management and followup of differentiated thyroid cancer. Debate still exists with regard to the optimal surgical approach but trends toward a more conservative approach, such as lobectomy, are being more favored, especially in papillary thyroid cancer, of tumor sizes less than 4 cm, in the absence of other high-risk suggestive features. Survival of patients with well-differentiated thyroid cancer was adversely affected by lymph node metastases. Prophylactic central LN dissection did improve accuracy in staging and decrease postop TG level, but it had no effect on small-sized tumors. Conservative approach was more applied with regard to the need and dose of radioiodine given postoperatively. There have been several advancements in the management of radioiodine resistant advanced differentiated thyroid cancers. Appropriate followup is required based on risk stratification of patients postoperatively. Many studies are still ongoing in order to reach the optimal management and followup of differentiated thyroid cancer.

## 1. Incidence and Prevalence of Thyroid Cancer

Thyroid cancer is one of the most common endocrine malignancies currently present. The estimated new thyroid cancer cases in the United States in 2012 are 56,460 and there are around 1780 deaths from thyroid cancer [1]. Incidence of thyroid cancer has been increasing. This could be related to the earlier detection of thyroid cancer with the current use of imaging and the use of FNA of all suspicious thyroid nodules. It is important to note that the overall 10-year mortality for DTC is low at about 7% but the recurrence rate occurrence is higher, causing considerable anxiety among patients and treating physicians. The current paper focuses on the controversies in the initial management and subsequent followup of well-differentiated thyroid cancer.

## 2. Pathogenesis of Differentiated Thyroid Cancer

Papillary and follicular thyroid carcinomas are the two histological subtypes of differentiated thyroid cancer. Both are indolent and have good prognosis overall. The biological behavior of these two carcinomas differ significantly, where papillary thyroid carcinoma is known to frequently metastasize to regional lymph nodes, whereas follicular thyroid carcinoma more frequently metastasizes to distant organs such as the lung, bone, and brain. Pathogenesis of differentiated thyroid carcinoma is multifactorial with both genetic and environmental factors playing an important role. For unknown reasons, it was found to be 2–4 times more common in women. Previous exposure to ionizing radiation including external irradiation of the neck would increase the incidence of thyroid cancer especially the papillary type. It was noted that there is a five- and two-fold increase of thyroid cancer incidence in obese men and women, respectively. In areas with adequate iodine intake differentiated thyroid carcinoma accounts for more than 80% of cases of thyroid cancer with the papillary type being the most common. In iodine deficient area there is a relative increase in the incidence of follicular and anaplastic thyroid cancer [2].

In recent years, the molecular basis of thyroid carcinogenesis has been investigated. In papillary thyroid carcinoma, BRAF mutations account for 45% of the cases with a higher prevalence in the “tall cell” differentiated forms. RET/PTC rearrangement was also found to account for 25–30% of papillary thyroid carcinoma cases. Point mutations of Ras gene and PAX 8/PPAR_*γ*_ rearrangement account for the majority of follicular thyroid carcinomas. Distant metastasis at the time of diagnosis was the most important prognostic factor for both papillary and follicular thyroid carcinomas. Extrathyroidal extension and lymph node metastasis were important prognostic factors for papillary thyroid carcinoma while the grade of invasiveness and carcinoma differentiation were important to evaluate the biological behavior of follicular thyroid cancer [3].

## 3. Staging of Differentiated Thyroid Cancer 

Cancer staging is an essential prognostic and integral part of cancer management. 17 different staging systems were described for patients with thyroid carcinoma [5]. The most currently used is the 6th edition TNM (tumor, node, and metastasis) staging, proposed by The American Joint Committee on Cancer (AJCC) and the International Union against Cancer Committee (UICC). Patients whose age is less than 45 years can be either Stage I or II with the only difference between the two stages is the absence or presence of metastasis. However, in older patients, nodal metastasis would classify those patients as stage III patients while distant metastasis would classify them as stage IV [6, 7]. It is important to note that the AJCC TNM classification to define thyroid cancer was recently criticized because it was considered that it may be too optimistic to classify younger patients this way. Effect of age and disease extent on mortality was examined in the AJCC staging system using survival data obtained from SEER Program from 1973 to 2005, with the aim of determining whether risk stratification did portray outcomes of DTC for young patients specifically. In multivariate analysis, 50% increased mortality was found for every decade from age 40 to 90, with this effect being more pronounced in women. When TNM staging applied for those above, the age of 45 years was applied to those less than 45 years and it was noted that there was an increased mortality for those now reclassified as stage III or IV based on LN involvement and tumor spread outside the thyroid but without distant metastases. However, the limitations of this study was that it did not analyze recurrence which is, of course, something more frequent than mortality in DTC [8]. Other important staging systems include the AGES (Age, Grade, Extent, Size) [9], the MACIS (Metastasis, Age, Completeness of resection, Invasion, Size) [10], both proposed by the Mayo Clinic, and both stratify patients into four risk groups, and the AMES (Age, Metastasis, Extension, Size) [11] proposed by the Lahey clinic, in addition to many other important staging systems as well. It was shown that after 20 years of diagnosis of differentiated thyroid cancer, the two most important prognostic factors were male gender and having the follicular type of thyroid cancer [12].

## 4. Controversies in the Management of Differentiated Thyroid Cancer

Initial management of differentiated thyroid cancer consists of thyroidectomy and, in certain cases, radioactive iodine therapy to ablate remnant remaining tissue and perhaps metastatic cancer. These are generally followed by long-term therapy with thyroxine with the aim of reducing circulating levels of thyrotropin (thyroid stimulating hormone, TSH) below normal. Revised American thyroid association guidelines were published in 2009 that looked at optimal management and follow up of differentiated thyroid cancer [13]. However, there are still controversies involving the optimal extent of initial surgery, the indications for prophylactic central lymph node dissection, the need for radioiodine therapy and the dose required, the degree of TSH suppression and its importance, and the aid of molecular markers to determine the risk of malignancy especially for indeterminate FNA samples.

### 4.1. Proper Extent of Initial Surgery

Extent of disease does affect outcome in patients with papillary thyroid carcinoma. Conflicting data exists in the literature regarding the optimal surgical approach for patients with differentiated thyroid cancer due to the fact that there are still no prospective, randomized studies addressing the issue specifically. ATA revised guidelines in 2009 stated that “for patients with thyroid cancer >1 cm, the initial surgical procedure should be a near-total or total thyroidectomy unless there are contraindications to this surgery. Thyroid lobectomy alone may be sufficient treatment for small (<1 cm), low-risk, unifocal, intrathyroidal papillary carcinomas in the absence of prior head and neck irradiation or radiologically or clinically involved cervical nodal metastases” [13]. Similarly, the 2007 British Guidelines for the management of thyroid cancer mentioned that “patients with a papillary thyroid cancer (PTC) more than 1 cm in diameter or with high-risk follicular thyroid cancer (FTC) should undergo near-total or total thyroidectomy”. However, it was mentioned that “patients with low-risk PTC and even FTC ≤1 cm in diameter may be treated with thyroid lobectomy alone” [14]. A study looked at whether total thyroidectomy, as compared to lobectomy, would result in decreased recurrence and improved long-term survival in 52,173 patients who underwent surgery for papillary thyroid carcinoma. 82.9% of those patients underwent total thyroidectomy while 17.1% underwent lobectomy. A 10-year recurrence rate was 4.6% in tumors <1 cm, 7.1% in 1.0–1.9 cm, 8.6% in 2.0–2.9 cm, 11.6% in 3.0–3.9 cm, 17.2% in 4.0–7.9 cm, and 24.8% in tumors >8.0 cm. For those patients whose tumor size was less than 1 cm extent of surgery had no impact on recurrence or survival, while for those patients with tumor size more than 1 cm lobectomy resulted in higher risk of recurrence and death (*P* = 0.04) [15]. Another retrospective review of 289 patients, selected for either thyroid lobectomy (*n* = 72) or total thyroidectomy (*n* = 217), without radioactive iodine remnant ablation, and followed by a single experienced endocrinologist with modern disease detection tools, in a tertiary referral center, was developed at the Memorial Sloan- Kettering Cancer Center (MSKCC). After a 5-year followup, disease recurrence was detected in 2.3% (5/217) of patients treated with total thyroidectomy, without radioactive iodine remnant ablation, and in 4.2% (3/72) of patients treated with thyroid lobectomy alone. Size of the primary tumor, presence of cervical lymph node metastases and American Thyroid Association risk category were all statistically significant predictors of recurrence. Changes in serum thyroglobulin were not helpful in identifying persistent/recurrent structural disease presence. In addition it was found that 88% (7/8) of patients who had recurrent disease were rendered clinically disease-free with additional therapies [16]. A recent study, utilizing the Surveillance, Epidemiology, and End Results program database of the National Cancer Institute, questioned the validity of the most recent ATA guidelines regarding the extent of surgery for papillary thyroid cancer. This study included 22,724 patients with papillary thyroid cancer, of which 5964 patients underwent lobectomy with a median followup of 9 years. Multivariate analysis showed no survival difference between patients who underwent total thyroidectomy versus lobectomy for all tumor sizes (<1 cm, 1–1.9 cm, 2–2.9 cm, 3–3.9 cm, and >4 cm. It was also shown that increased age, increased tumor size, extrathyroidal extent, and positive nodal status displayed significantly worse disease specific survival and overall survival (*P* < 0.001 [17]). The debate regarding the extent of surgery in patients with papillary thyroid carcinoma persists and a well-designed prospective randomized study is needed in order to clarify whether less strict approach should be applied in such cases even for those patients whose tumor size is more than 1 cm.

### 4.2. Prophylactic versus Therapeutic Central LN Dissection

Presence of LN metastasis has a negative outcome in general and can be identified preoperatively or during operation. Prophylactic neck dissection is defined as the removal of seemingly normal lymph nodes apparent as per ultrasound and during surgery. Prophylactic central LN dissection would improve accuracy in staging, decreases postoperative TG level, can be performed safely as total thyroidectomy alone in experienced hands leading sometimes to avoidance of further operations in central neck, and may lead to lower recurrence and improved mortality. The other rationale to perform prophylactic central LN dissection is the questionable ability of the preoperative ultrasound or intraoperative assessment to adequately evaluate the central neck compartment [18–20]. However, it may lead to a higher rate of hypoprathyroidism that was reported in one study to occur in up to 14% of patients after prophylactic central LN dissection as opposed to 1-2% after total thyroidectomy, and the higher possibility of recurrent laryngeal injury, with the absence of level 1 data of lower recurrence and improved survival [18]. Hence, a current widely debated subject in PTC patients is whether prophylactic central neck dissection (PCND) should be done in patients who are found to have clinically negative nodes by ultrasound, on physical exam, and during intraoperative assessment.

Some studies have shown no difference in survival in those patients who underwent lymphadenectomy versus those who did not [21]. On the other hand, a large multi-institutional study looked of how the presence of lymph node disease would adversely affect the outcome in 19,918 patients with papillary and follicular thyroid carcinomas using Surveillance, Epidemiology, and End Results (SEER) database, which is a large scale sample of 14 percent of the U.S. population. Using a multivariate analysis it was shown that those patients whose age is more than 45 years, who had the presence of distant metastasis, tumors of large size size >4 cm, and lymph node metastasis did significantly have predicted poor outcome. Overall survival at 14 years was 82 per cent for patients with node negative as opposed to 79 per cent for node positive patients (*P* < 0.05); hence, survival of those patients with well-differentiated thyroid cancer was adversely affected by lymph node metastases where they were at greater risk of recurrence and death [22]. Moreover, a retrospective study was done over a period of 12 month, which included patients before year 2002, when the initial management included total thyroidectomy and radioiodine therapy for papillary thyroid cancer, and patients managed after the year 2002, where management was modified to include prophylactic central LN dissection in addition to total thyroidectomy. Those patients with regional LN metastasis had higher stimulated thyroglobulin levels at one year followup if no prophylactic central LN dissection was done. In addition, more complete lymphadenectomy was associated with a lowering of thyroglobulin levels overall [23].

Recently in a study including 115 patients an aggressive approach to papillary thyroid cancer was adapted, with prophylactic bilateral level dissection of LNs (level VI) and in some cases, ipsilateral symmetrical levels II, III, and IV dissections, in patients with small papillary thyroid cancer, who had negative preoperative neck ultrasound. Mean size of PTC was 12.5 mm and extension of tumor beyond thyroid capsule was found in 29% of patients, with central LNs found to be involved in 45% of patients, and lateral LNs involved in 47% of patients. 58% of patients underwent radioiodine therapy afterwards, in those PTC tumors larger than 18 mm with lymph node metastases, or having aggressive histology and younger age (<18 years). Prophylactic lymph node dissection modified the indication for radioiodine ablation in 30% of patients. At 1-year followup all patients had negative neck ultrasound examinations, and 97% had an undetectable stimulated serum thyroglobulin [24].

These opposing findings in retrospective studies continue the current and do not provide a uniform approach in the management of PTC; however, a current prospective multicenter clinical trial has been submitted to the National Institute of Health trying to look at the benefits and risks of an ipsilateral prophylactic central LN dissection. ATA guidelines consider prophylactic central LN dissection in patients with T3 and T4 tumors but not in those who has smaller tumors [13]. The Japanese association of endocrinology recommends prophylactic central LN dissection based on clinic-pathological features rather than treating all patients uniformly in the same way [25]. 

### 4.3. Radioactive Iodine Ablation

In addition to the current controversy regarding the extent of surgery and the need for prophylactic central LN dissection, the need for postoperative radioactive iodine (RAI) ablation is also a current widely debated subject. RAI therapy is used as an adjunct to surgery, to eradicate occult persistent or metastatic disease, with the aim of reducing the risk of recurrence and improving mortality. Differentiated thyroid cancer, especially papillary thyroid cancer, has an excellent prognosis overall, that is why the need for postoperative RAI therapy has been questioned. As per ATA guidelines, RAI is recommended in patients with gross extrathyroidal extension, known distant metastases, and for tumors more than 4 cm. For patients whose tumors are 1 to 4 cm, a selective approach is recommended. RAI is not recommended for tumors less than 1 cm even if multifocal but micro-PTC and no high-risk features present [13]. Some even advocate no RAI ablation for well-differentiated PTC for tumors between 1 and 4 cm who have less than 3–5 metastatic cervical lymph nodes that are less than 5 mm in diameter [26]. On the other hand, British guidelines mentioned that “majority of patients with a tumor more than 1 cm in diameter, who have undergone a near-total/total thyroidectomy, should have 131I ablation” [14]. A recent retrospective study revisited the issue of RAI ablation and analyzed 289 patients, of which 74% were low risk and 26% were intermediate risk according to the ATA risk stratification study, from the Memorial Sloan-Kettering Cancer Center. 75% of patients were treated with total thyroidectomy and 25% treated with lobectomy. Selective central neck lymph node dissection was done in 5% of those patients. It was shown that only 2% of those treated with total thyroidectomy and 4% with lobectomy who did not have RAI therapy recurred. Even when tumors >1 cm were only analyzed, the recurrence rate following total thyroidectomy without RAI remained low at 4% [16].

With regards to the dose of RAI needed, larger doses of RAI therapy (100–200 mCi) are considered appropriate, if residual microscopic disease is suspected, or aggressive tumor histology detected. As for distant metastatic, disease a “fixed-dose” regimen is the most widely used, with 150 mCi for cervical and higher doses in the range of 200–250 mCi for pulmonary metastases or skeletal metastases [27]. As for the optimal dose of RAI therapy needed in those patients at low risk, a recent randomized phase 3 trial compared two thyrotropin stimulation methods (thyroid hormone withdrawal and the use of recombinant human thyrotropin) and two different radiodine doses (I131) (1.1 GBq and 3.7 GBq), in a 2-by-2 design, in 752 patients with low-risk differentiated thyroid cancer, where patients were included if they had pT1 (tumor diameter ≤ 1 cm) and N1 or Nx, pT1 (with tumor diameter >1 to 2 cm) and any N stage, or pT2N0 with absent distant metastasis. Thyroid ablation was equivalent between different I131 doses and between different thyrotropin stimulation methods [28]. Another recent study showed the same outcome as the previous study where patients aged 16 to 80 with T1 to T3 tumor size, with possible spread to LN, but no metastasis, underwent low- versus high-dose RAI, in combination with either TSH alfa or TH withdrawal prior, and it was shown that the success rates were comparable in all groups of patients ranging between 85 and 89% [29].

### 4.4. Molecular Markers to Aid in Malignancy Diagnosis in Indeterminate FNA Samples

The expansion of knowledge regarding genetic mutations in thyroid cancers has led to the use of molecular markers as an indicator of prognosis and for the malignancy diagnosis especially in indeterminate FNA samples (BRAF, RAS, RET/PTC, and PAX8/PPAR*γ*) [2]. Recent large prospective studies have emphasized the ability of genetic markers (BRAF, RAS, RET/PTC, and PAX8/PPAR*γ*) and protein markers (galectin-3) to significantly improve and affect the preoperative diagnostic accuracy especially for patients who have indeterminate thyroid nodules [30–34]. Moreover, the use of such molecular markers was further recommended by the 2009 revised ATA guidelines where the sensitivity of malignant diagnosis in FNA thyroid nodules was found to be increased from 44% to 80%, when comparing cytology alone to cytology combined with molecular testing for the markers in most of those studies. One of those studies showed that the sensitivity of malignant diagnosis in FNA increased from 60%, with cytology alone, to 90% with cytology and molecular testing for BRAF, RAS, RET, TRK, and PPAR*γ* mutations [33]. It can be concluded that the best current preoperative tool for thyroid cancer is the cytological examination of molecular markers of FNA biopsies from indeterminate thyroid nodules.

## 5. Controversies in the Followup of Differentiated Thyroid Cancer

### 5.1. Role of TSH Suppressive Therapy

Thyroid hormone suppression is now a recognized treatment for patients with differentiated thyroid cancer. In 2002, the first meta-analysis was published regarding TSH suppressive therapy but included 10 studies with small series of patients. This meta-analysis concluded that treatment with high thyroxine doses was effective in decreasing recurrence but it had little importance with regards to the overall survival [35]. Then in 2006, a study stratified the effect of this therapeutic approach and found that this approach had no effect on survival in stage I low-risk patients, but patients staged II, III, and IV did have worse survival when TSH level was maintained more than 3 mU/L [36]. The revised ATA guidelines state that “in patients with persistent disease, the serum TSH should be maintained below 0.1 mU/L indefinitely in the absence of specific contraindications.” They also added that “in patients who are clinically and biochemically free of disease but who presented with high-risk disease, consideration should be given to maintaining TSH suppressive therapy to achieve serum TSH levels of 0.1–0.5 mU/L for 5–10 years” but “in patients free of disease, especially those at low risk for recurrence, the serum TSH may be kept within the low normal range (0.3–2 mU/L). They further mentioned that “in patients who have not undergone remnant ablation who are clinically free of disease and have undetectable suppressed serum Tg and normal neck US, the serum TSH may be allowed to rise to the low normal range (0.3–2 mU/L)” [13]. Moreover, British guidelines recommended that in those patients being treated with 131I, levothyroxine therapy should be added three days later, with the aim of suppressing serum thyroid-stimulating hormone (TSH) to <0.1 mIU/L. In patients confirmed to be low risk, a serum TSH <0.5 mIU/L was considered acceptable [14]. It is important to mention that the only randomized prospective study conducted to date, recently published, assessed the efficacy of thyroid hormone suppressive therapy, where they randomized 400 patients undergoing surgery for DTC into a group treated with thyroxine to achieve TSH suppression, while other group of patients treated to maintain TSH within the normal range. The authors found that after a mean followup of 7 years, there was no significant difference between the two groups in regard to disease-free time, relapse, and time of relapse, distant metastases, overall mortality, or specific mortality [37]. In addition it was noted that TSH suppressive therapy was not without risk. A recent review noted increased incidence of kidney, pancreas, ovarian, and breast cancers [38]. Large epidemiological studies, including 29,000 patients, in Norway, monitored for 9 years after having suppressed TSH levels to less than 0.5 Mu/L, noted increased cancer incidence (hazard ratio 1.34) [39]. It was postulated that integrin activation by THs was responsible for promoting angiogenesis by thyroid hormones through the activation of mitogen-activated protein kinase (or MAPK pathway) [40].

Therefore the beneficial role of TSH suppressive therapy is questioned, given the recent randomized study that failed to show any benefit for TSH suppressive therapy, in addition to its possible overall increased risk to other cancer developments.

### 5.2. Diagnostic Imaging for the Followup of DTC

The 3-to-12-month monitoring of patients with extrathyroidal invasion or local-regional nodal metastases using ultrasound of neck and serum thyroglobulin should be performed on all patients with differentiated thyroid cancer as per ATA guidelines [13]. It is worth noting that sometimes elevated thyroglobulin or thyroglobulin antibody is found along with negative radioactive iodine scan and in such cases ultrasound is definitely recommended and this could be indicative of nonradioiodine avid residual or recurrent disease. It was found that half of the patients with negative radioiodine scan have recurrent disease based on the use of ultrasound, which can accurately identify lesions in the neck even as small as 3 mm [41]. Other imaging techniques that can be used in followup of certain patients include CT scan of the neck with IV contrast, CT scan of the chest, and magnetic resonance imaging (MRI) although the sensitivity of these imaging techniques are inferior to ultrasound, and they are frequently used if ultrasound is not available, or if deep posterior disease suspected. Fluorodeoxyglucose positron-emission tomography (FDG-PET or PET)/CT imaging is useful for the detection of radioiodine negative and thyroglobulin positive thyroid cancer which tends to have higher glucose metabolism and this points to tumor dedifferentiation [42, 43]. TSH stimulates 18FDG uptake by differentiated thyroid carcinoma making it more sensitive. However, a large multicenter study showed that rh TSH stimulated PET-CT changed treatment plan in only 6% of cases [44].

### 5.3. Long-Term Followup and Stratification of DTC Patients

As shown in Table 1 Patients can be risk stratified at 6 months followup based on their unstimulated and stimulated thyroglobulin level, thyroglobulin antibodies, neck exam, neck ultrasound, diagnostic radioiodine scans, use of other cross-sectional imaging like CT scans, MRI, or PET into having excellent, acceptable, and incomplete response [4].

Patients who are considered to have excellent response based on undetectable thyroglobulin including stimulated thyroglobulin, normal neck ultrasound, negative radioiodine scan, and negative scans (CT, MRI, or PET) can be followed with yearly physical exam and yearly suppressed thyroglobulin with no need for stimulated thyroglobulin. Patients who had acceptable response, meaning detectable suppressed thyroglobulin < 1 ng/mL, stimulated thyroglobulin < 10 ng/mL, nonspecific changes in neck ultrasound including few sub-centimetric LNs, and negative radioiodine scans, can be followed with yearly physical exam, suppressed and stimulated thyroglobulin yearly for at least another 3 years. Patients who have incomplete response, due to increased suppressed TG > 1 ng/mL, increased stimulated thyroglobulin > 10 ng/mL with cervical LNs > 1 cm, positive radioiodine scan, and positive other imaging modalities including CT, MRI, or PET, should be referred for additional therapies [4]. This was clearly shown in Table 2. On the other hand, based on expert opinion, British guidelines mentioned that serum thyroglobulin should be checked in all postoperative patients with differentiated thyroid cancer six weeks after surgery and stimulated thyroglobulin indicated 6 months after 131I ablation. They also added that postablation whole-body scan, done after stopping levothyroxine for four weeks, should be considered 3–10 days after 131I ablation. However, in low-risk patients, the measurement of stimulated thyroglobulin without a diagnostic 131I WBS may be adequate. In such cases, it was recommended that ultrasonography of the neck 6–12 months after thyroidectomy is indicated. As for the long-term recurrence, proposed by the British guidelines, annual clinical examination, annual measurement of serum Tg and TSH, diagnostic imaging and FNA as indicated are required [14].

### 5.4. Management of Advanced or Metastatic Thyroid Cancer

Surgery in advanced thyroid carcinomas is the best modality used for recurrent neck metastases, since most recurrences are seen in the thyroid bed or regional lymph nodes. This is usually followed by further radioactive iodine, especially if the recurrence is radioiodine avid, along with thyroid hormone suppression. If the gross tumors are not radioactive iodine avid then further postoperative radioactive iodine will be of limited benefit. Surgery is also considered for isolated metastases in bone and brain. External beam radiotherapy (EBRT) may be an effective adjuvant therapy in patients with locally invasive papillary carcinoma who are 45 years of age and older [45, 46]. British guidelines noted that external beam radiotherapy can occasionally be used in patients with pT4 tumours (TNM staging) who have residual disease in the neck not amenable to surgery, especially in tumours not taking up 131I. In addition it was found to have a possible role as a palliative measure in patients with advanced local or distant disease [14]. Patients who are found to have brain metastasis can be treated with surgery which was found in one study to improve median survival from four to 22 months in patients who had 1 or more brain metastases. Radioiodine therapy and/or external beam radiotherapy should be considered after surgical resection with the concomitant use of steroids. External beam radiation therapy (EBRT) or gamma knife radiosurgery and IV bisphosphonates has also been used for patients with painful bony metastasis [47, 48]. Clinical trials should be considered the first-line therapy for patients who are considered to have nonavid radioactive iodine response. Systemic chemotherapy has been tried in widespread progressive disease that is radioiodine resistant but has not been shown to be effective to date [49]. Several tyrosine kinase inhibitors have been tried until now with a partial response ranging between 13% and 50% [50–52].

## Figures and Tables

**Table 1:** Variables determined during follow-up that predict response to therapy (Tg→ thyroglobulin) (adapted from [4]).

	Excellent response	Acceptable response	Incomplete response
Suppressed Tg	Undetectable	Detectable but <1 ng/mL	>1 ng/mL
Stimulated Tg	Undetectable	<10 ng/mL	>10 ng/mL
Trend in suppressed Tg	Remains undetectable	Declining	Stable or rising
Anti-Tg antibodies	Absent	Absent or declining	Persistent or rising
Neck examination	Normal	Normal	Palpable disease
Neck ultrasonography	No evidence of disease	Nonspecific changes in thyroid bed, Stable millimeter sized cervical LN even if abnormal by US criteria	Evidence of structurally significant recurrent/persistent disease in the thyroid bed (>1 cm), cervical lymph nodes (>1 cm), or distant metastases, particularly if structurally progressive or FDG avid
Diagnostic RAI WBS	No evidence for RAI avid disease	No evidence for RAI avid disease, very faint uptake in thyroid bed only	Persistent/recurrent RAI avid disease present
Cross-sectional imaging (MRI, CT)	No evidence of disease	Non-specific changes	Structural disease present
FDG PET scanning	No evidence of disease	Non-specific changes consistent with normal variants or inflammatory changes	FDG avid disease present

**Table 2:** Follow-up strategy based on risk groups (adapted from [4]).

	Initial estimate of risk of recurrence first 2 years of followup incomplete response		
Suppressed Tg	Low risk Q 6 months	Intermediate risk Q 6 months	High risk Q 6 months
Stimulated Tg	Not required	<10 ng/mL	>10 ng/mL
Neck ultrasound	Q year × 2	Q year × 2	Q year × 2
Diagnostic RAI WBS	Not required	1-2 years	1-2 years
Cross-sectional imaging (MRI, CT)	Not required	Not required	If Tg elevated or high clinical suspicion

Secondary risk stratification response to therapy assessment			

Ongoing followup	Yearly physical examination, yearly suppressed Tg	Yearly physical examination, yearly suppressed Tg, stimulated Tg to document undetectable Tg on suppression, continued observation/assessment of indeterminate structural abnormalities for at least another 2-3 years	Consider additional cross-sectional imaging, possibly FDG PET scan and the need for additional therapy
